# Pain as relief

**DOI:** 10.1192/j.eurpsy.2021.659

**Published:** 2021-08-13

**Authors:** P. García Vázquez, R. Gomez Martinez

**Affiliations:** Psiquiatría, Complejo Asistencial Universitario León, León, Spain

**Keywords:** adolescence, Cryothermic dermatitis artefacta, Pain

## Abstract

**Introduction:**

Dermatitis artefacta (DA) is a condition whereby self-induced skin damage is the means used to satisfy a desire to assume the sick role.

**Objectives:**

To describe clinical evaluation, diagnosis, treatment and evolution of an 15 years-old woman with DA.

**Methods:**

Retrospective review of clinical records, including dermatology, psychiatry and Pathology.

**Results:**

A 15-year-old woman, who come to the Child Psychiatry consultations derived by the Dermatology Service, which is attended by the appearance of multiple bullous lesions throughout the body. Since August, the patient has reported a worsening of her mood, with feelings of loneliness and vital emptiness, with somatic and psychic anxiety referred. In the Dermatology office, she does not recognize self-infliction and the patient is derived to psychiatry consultation and solicited a skin biopsia. In the psychiatric interview, she recognizes that burns occur with a deodorant spray. She admitted doubts about her sexuality for months, claiming to be homosexual for the first time. When she burns, feels pleasure and relief. At the exploration: She wears a gay pride flag bracelet. Expressionless attitude. Cold contact. Approachable. Slightly collaborative. Without major affective disorders. No somatic anxiety, nor psychic. Short speech, impoverished language. Personality traits Cluster B and C. •Salamanca Questionnaire: Dependent personality, and in the background anxiety and histrionic. •Plutchik Impulsivity Scale: 14. •Toronto Alexithymia Scale: 64

**Conclusions:**

The prognosis of the condition is variable, but it has been shown that resolution of the underlying psychosocial stressor leads to improvement. Multinucleated keratinocytes, a pathognomonic lesion of cryodermatitis artefacta, are discovered in the Pathology.

